# The military gear microbiome: risk factors surrounding the warfighter

**DOI:** 10.1128/aem.01176-23

**Published:** 2024-01-03

**Authors:** Car Reen Kok, Zakariae Bram, James B. Thissen, Timothy S. Horseman, Keith S. K. Fong, Susan A. Reichert-Scrivner, Carmen Paguirigan, Kelsey O'Connor, Kristina Thompson, Alexander E. Scheiber, Shalini Mabery, Viseth Ngauy, Catherine F. Uyehara, Nicholas A. Be

**Affiliations:** 1 Physical and Life Sciences Directorate, Lawrence Livermore National Laboratory, Livermore, California, USA; 2 Tripler Army Medical Center, Honolulu, Hawaii, USA; 3 School of Medicine, Uniformed Services University of the Health Sciences, Bethesda, Maryland, USA; Centers for Disease Control and Prevention, Atlanta, Georgia, USA

**Keywords:** combat injury, military medicine, microbial genomics, gear microbiome

## Abstract

Combat extremity wounds are highly susceptible to contamination from surrounding environmental material. This bioburden could be partially transferred from materials in immediate proximity to the wound, including fragments of the uniform and gear. However, the assessment of the microbial bioburden present on military gear during operational conditions of deployment or training is relatively unexplored. Opportunistic pathogens that can survive on gear represent risk factors for infection following injury, especially following combat blasts, where fibers and other materials are embedded in wounded tissue. We utilized 16S rRNA sequencing to assess the microbiome composition of different military gear types (boot, trouser, coat, and canteen) from two operational environments (training in Hawai’i and deployed in Indonesia) across time (days 0 and 14). We found that microbiome diversity, stability, and composition were dependent on gear type, training location, and sampling timepoint. At day 14, species diversity was significantly higher in Hawai’i samples compared to Indonesia samples for boot, coat, and trouser swabs. In addition, we observed the presence of potential microbial risk factors, as opportunistic pathogenic species, such as *Acinetobacter*, *Pseudomonas,* and *Staphylococcus*, were found to be present in all sample types and in both study sites. These study outcomes will be used to guide the design of antimicrobial materials and uniforms and for infection control efforts following combat blasts and other injuries, thereby improving treatment guidance during military training and deployment.

**IMPORTANCE:**

Combat extremity wounds are vulnerable to contamination from environments of proximity to the warfighter, leading to potential detrimental outcomes such as infection and delayed wound healing. Therefore, microbial surveillance of such environments is necessary to aid the advancement of military safety and preparedness through clinical diagnostics, treatment protocols, and uniform material design.

## INTRODUCTION

Opportunistic pathogens persist in the environment and become infectious during host perturbation under conditions favorable to the organism (1). These pathogens include commensal species such as skin-associated *Staphylococcus aureus* and environmental species such as *Pseudomonas aeruginosa* (2, 3). As such, bioburden surveillance is commonly exercised in hospital environments to prevent the transmission of nosocomial pathogens among patients and healthcare workers (4). Similar bioburden threats exist in military operational environments under both training and deployed circumstances. Opportunistic pathogens, when present at the point of injury, can invade high surface area wounds and increase the risk of contamination. Even species rarely associated with active infections in healthy civilian populations hold the potential to be problematic in a combat environment. Such injuries are known to harbor atypical pathogens (5), and extreme polytrauma can result in immune dysfunction (6, 7). Moreover, military clothing has also been shown to significantly impact injury patterns in gunshot wounds (8).

Aligned with previous reports on the contamination of combat wounds with soil, clothing, and skin (9, 10), foreign materials, such as fragments of uniform and gear, can act as a source of pathogenic bioburden. In fact, nosocomial pathogens such as *Enterococcus faecium*, *Staphylococcus aureus*, *Pseudomonas aeruginosa*, and *Acinetobacter baumannii* have been shown to survive on different textiles for prolonged time periods, acting as a probable source of pathogen transmission in hospitals (11–13). Recent studies have shown that these nosocomial pathogens are among the species with the highest occurrence of contamination in wound combat tissue injury (14).

While a potential source of infection, there have been few reports on the microbial bioburden present on military uniforms. A study in the 1980s demonstrated the successful culturing of pathogenic species such as Staphylococci, Streptococci, and Clostridia from Finnish military field uniforms, which were subsequently found to contaminate projectile wounds in an animal model (15).

More recently, a survey of microbial contamination on military and civilian uniforms was carried out in the emergency department of a military hospital (16). While no difference in microbial load was observed between the two uniform groups, elevated bio-behavioral risk factors such as poor handwashing hygiene and reduced laundering practices were reported in the military uniform group. However, these pathogen surveillance efforts were carried out using traditional culture methods, which are less effective in capturing whole microbial communities and exclude especially fastidious or unculturable bacteria. In this study, we attempt to bridge this gap in knowledge and provide foundational data on the microbiomes of military gear through metagenomic sequencing and evaluate the potential bioburden threats surrounding military soldiers.

To achieve this goal, we obtained environmental swab specimens from the gear of soldiers from two independent military cohorts: participants in the Jungle Operations Training Course (JOTC) on Oahu, Hawai’i, and active-duty service members deployed to Indonesia. We gathered longitudinal samples across four different military gear types. The JOTC exercises include situational jungle and waterborne tactics and operations, thereby representing a correlate for an operational scenario in the context of the local environment on Oahu, Hawai’i. The military cohort in Indonesia represents a true deployed scenario and the extended associated operational requirements. Due to the low community biomass present on swab samples, 16S rRNA amplicon sequencing was used for taxonomic profiling. Overall, this study emphasizes the importance of microbial surveillance in environments of proximity to the warfighter to advance military preparedness and protect warfighter safety and wellbeing.

## RESULTS AND DISCUSSION

### Spatiotemporal shifts in microbial diversity and composition

**Fig 1:**
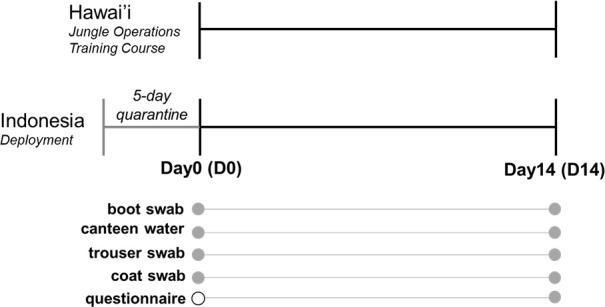
Study timeline and sample collection. Samples from four different gear types were collected from Hawai’i and Indonesia study sites across two different timepoints. Filled circles indicate samples that were collected.

**Fig 2:**
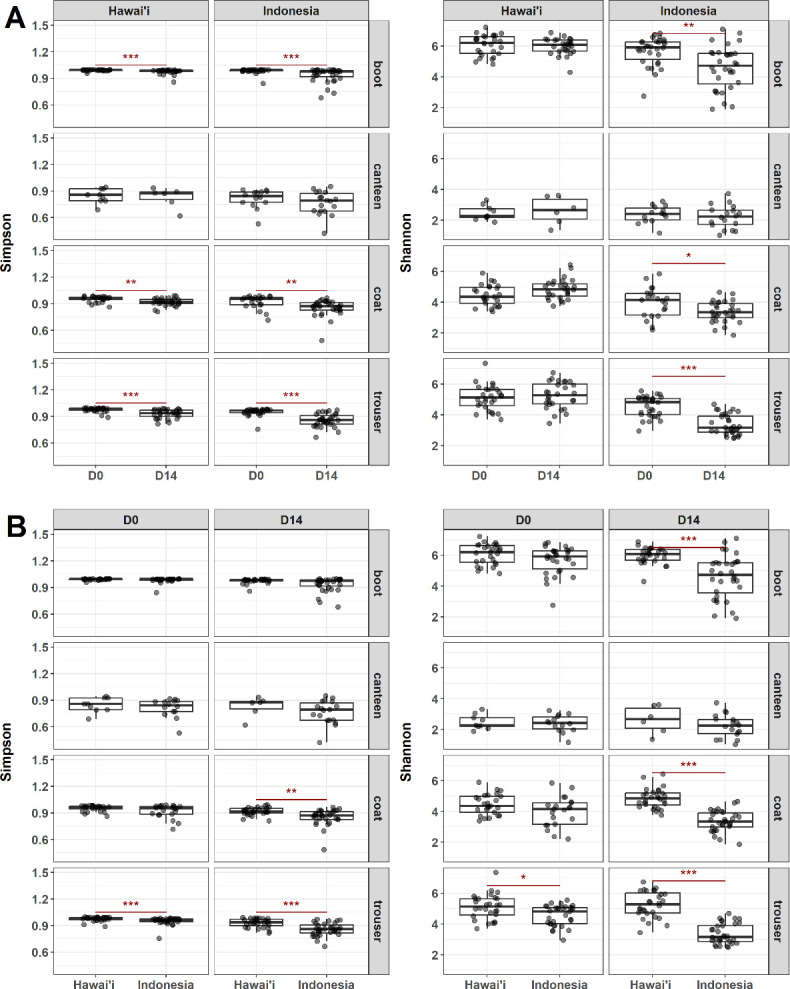
Alpha diversity of samples across gear type, study sites, and sampling timepoints. Alpha diversity was measured using the Simpson and Shannon indexes as diversity metrics. Alpha diversity was evaluated within each gear type between (A) timepoints for each study site and between (B) study sites for each timepoint. Significant differences were determined using Wilcoxon tests with FDR correction. *P < 0.05, **P < 0.01, and ***P < 0.001.

**Fig 3:**
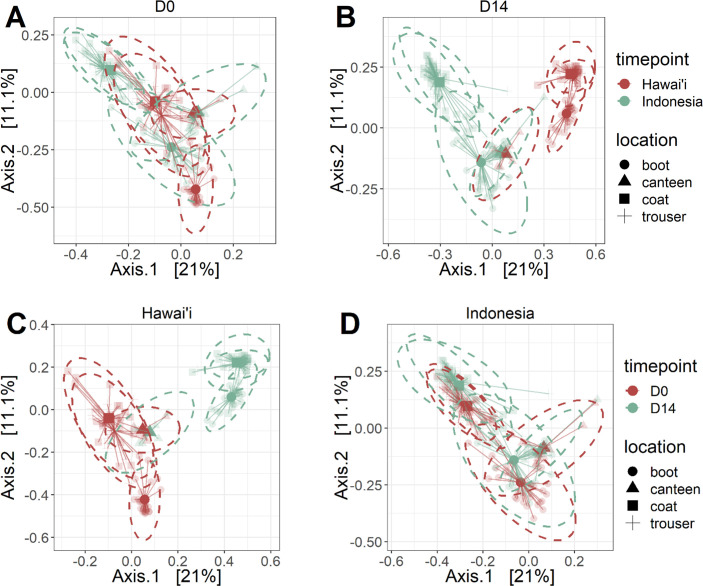
Beta diversity and microbial composition of gear samples. Beta diversity was measured and visualized in two-dimensional space using MDS of Bray-Curtis distances. Sample visualization is presented here based on (A and B) time and (C and D) study sites for comparisons. Samples between Indonesia and Hawai’i study sites were compared at (A) D0 and (B) D14. Colors represent study sites and symbols represent different gear types. Samples between timepoints were also compared for (C) Hawai’i and (D) Indonesia cohorts. Colors represent timepoints and symbols represent different gear types. Edges connect data points to centroids within a group (study location or timepoint location).

Gear specimens were collected from participants of the JOTC conducted on Oahu, Hawai’i, and active-duty service members stationed in Hawai’i participating in a military exercise in Indonesia (Fig. 1). Samples were taken at baseline (D0) upon arrival and 14 days after the start of operations (D14) for sequencing and microbiome analyses. Microbial diversity was evaluated and compared across gear types, study sites, and timepoints. Alpha diversity was measured using the Simpson and Shannon indexes capturing species richness and evenness (Fig. 2; Fig. S1A). Beta diversity was measured using Bray-Curtis distances. Compositional differences between samples and across sample types, study sites, and timepoints were determined using PERMANOVA. The resulting multidimensional scaling (MDS) plots were visualized in two-dimensional space (Fig. 3; Fig. S1B).

PERMANOVA analyses revealed significant effects of study sites, gear types, and timepoints along with significant interactions between all three factors (Table 1; *P* < 0.001). From the MDS plots, trouser and coat samples appear to harbor similar microbial communities regardless of the study site or timepoint, while the microbial communities of boot and canteen samples were distinct from other sample types (Fig. 3). The microbial composition of canteen samples appeared unchanged through time and was similar across study sites, while both Shannon and Simpson diversity of canteen samples were found to be significantly lower compared to all other gear types regardless of study location, timepoint, and diversity metric (Fig. S1A). Conversely, boot samples were the most diverse, followed by trouser and coat samples. The high microbial diversity of boot samples is likely driven by the presence of environmental taxa resulting from direct contact with the ground and the accumulation of microbes over time. In fact, previous studies have reported high similarities between shoe and floor microbiomes along with the presence of environmental species on shoes (17, 18).

**TABLE 1:** Three-factor PERMANOVA analyses based on Bray-Curtis distances^*a*^

Factors	Degrees of freedom	Sum of squares	*R* ^*2*^	*F*	Pr > *F*
Site	1	16.26	0.11	75.33	9.99e-5
Gear	3	19.36	0.13	29.9	9.99e-5
Time	1	9.48	0.06	43.9	9.99e-5
Site × gear	3	6.26	0.04	9.66	9.99e-5
Site × time	1	9.06	0.06	41.96	9.99e-5
Gear × time	3	3.73	0.03	5.76	9.99e-5
Site × gear × time	3	2.63	0.02	4.06	9.99e-5

^*a*^ Results from three-factor PERMANOVA analyses to determine significant factors (study site, gear type, and timepoint) alongside significant interactions.

Compositional shifts across time were more apparent for Hawai’i samples compared to Indonesia samples, reflecting temporal changes that differed depending on the study site (Fig. 3C and D). This is likely a result of early convergence during a mandatory 5-day quarantine period prior to the first sampling timepoint (Fig. 1). Comparing alpha diversity across timepoints further revealed that, except for canteen samples, Simpson and Shannon diversity decreased significantly from D0 to D14 for all Indonesia samples (Fig. 2A). Upon further investigation, we determined that soldiers in JOTC had limited access to laundry facilities and a more compressed training schedule compared to those in Indonesia, which may have influenced the observed reduced diversity in Indonesia samples. A significant decrease in the Simpson index was also observed for Hawai’i boot, coat, and trouser samples across time. In contrast, no significant differences in Shannon index were observed across time in Hawai’i samples. Discrepancies between both measures of diversity are likely attributed to the respective influence of species richness in Shannon index calculations and species evenness in Simpson index calculations.

Simpson and Shannon diversity of trouser samples from Hawai’i were significantly higher compared to Indonesia samples at D0, while Hawai’i coat and trouser samples had significantly higher Simpson and Shannon diversity compared to Indonesia samples at D14 (Fig. 2B). From the MDS plots, compositional differences between Hawai’i and Indonesia samples were also apparent at D14 (Fig. 3B). Together, these results suggest that the microbial communities that form on uniforms were highly dependent on sampling location as a result of convergence with its surrounding environments along with biobehavioral practices such as laundering frequency.

### Military gear-associated microbes and species abundances

**Fig 4:**
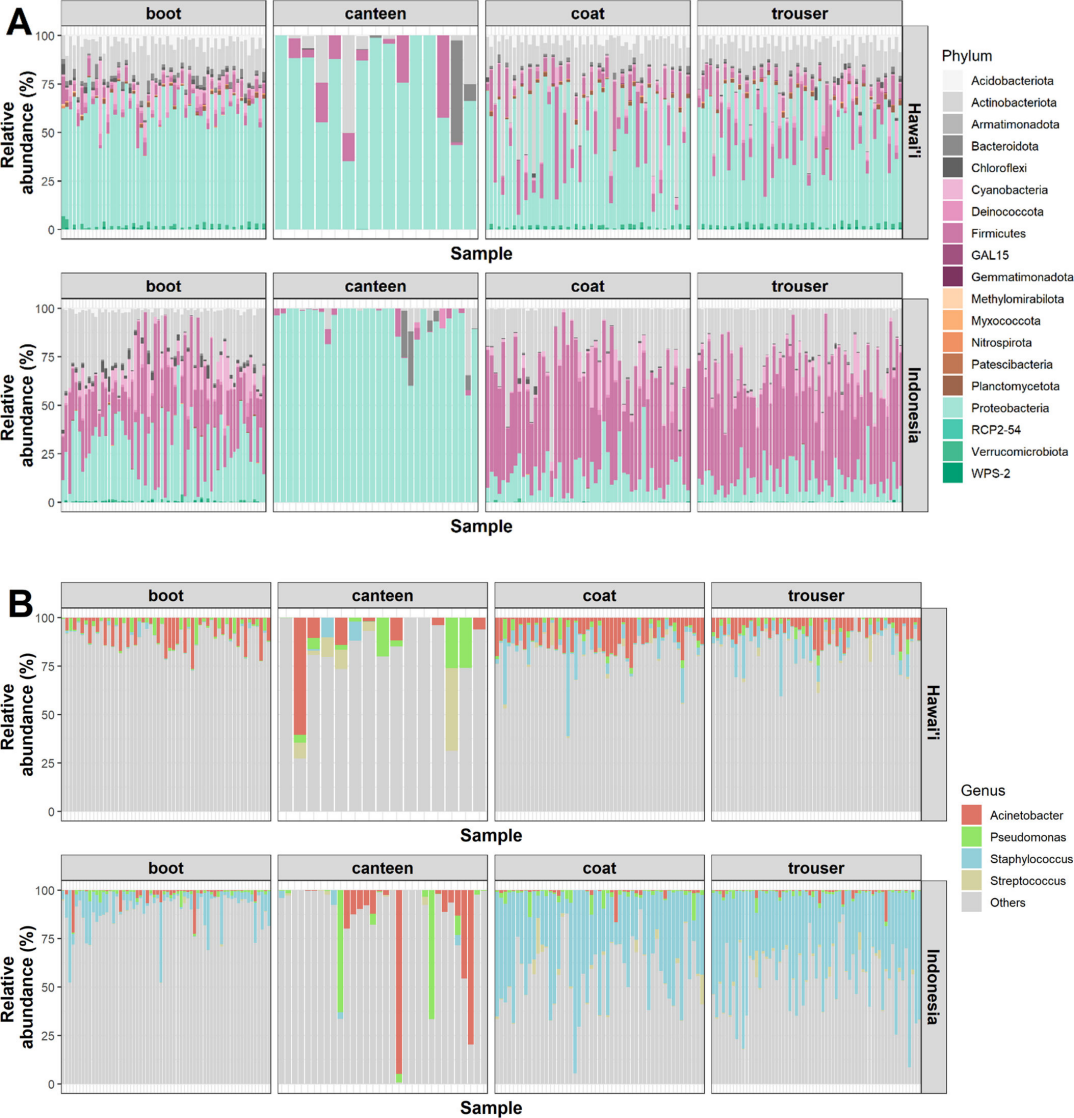
Relative abundances of phyla and genera across samples. Taxonomic abundances were represented at the (A) phyla and (B) genus levels across all samples. Samples were grouped according to gear type with each column representing a sample. Colors correspond to the different phyla and genera present in the samples.

The relative abundances of microbial species were examined across samples (Fig. 4; Fig. S3). The top abundant phyla across all samples include *Proteobacteria* (42.4% ± 28.5%), *Firmicutes* (25.1% ± 24.4%), *Actinobacteriota* (22.8% ± 16.3%), and *Cyanobacteria* (4.37% ± 7.17%) (Fig. 4A). On average, canteen samples were dominated by *Proteobacteria* (Indonesia: 92.9% ± 11.2% and Hawai’i: 78.6% ± 21.1%), including *Alphaproteobacteria* species such as *Brevundimonas, Sphingomonas,* and *Caulobacter* and *Gammaproteobacteria* species such as *Acinetobacter* and *Escherichia-Shigella* (Fig. S2 and S3). There was no indication of potential pathogens that might result in adverse health events upon consumption. Aside from canteen samples, the Indonesia samples appeared to harbor a relatively high percentage of *Firmicutes* (trouser: 52.4% ± 17.27%, coat: 50.7% ± 18.8%, and boot: 31.5% ± 24.3%), while the Hawai’i samples had high abundances of *Proteobacteria* (trouser: 52.5% ± 17.0%, coat: 49.5% ± 22.1%, and boot: 60.7% ± 8.3%) (Fig. 4A). Indonesia boot samples also had a high relative abundance of *Proteobacteria* (31.1%).

Further investigation at the genus level revealed the presence of genera associated with wound colonization and healing including *Proteobacteria* such as *Acinetobacter* (7.6% ± 7.9%: Hawai’i and 2.7% ± 9.6%: Indonesia) and *Pseudomonas* (2.1% ± 3.7%: Hawai’i and 2.0% ± 6.6%: Indonesia) and *Firmicutes* species such as *Staphylococcus* (3.6% ± 7.2%: Hawai’i and 25.1% ± 23.1% Indonesia) and *Streptococcus* (1.0% ± 3.7%: Hawai’i and 0.8% ± 2.2%: Indonesia) (Fig. 4B). Other *Firmicutes* and *Proteobacteria* species found in gear samples include *Exiguobacterium*, *Bacillus*, and *Aerococcus* (Fig. S3A) and *Enhydrobacter*, *Sphingomonas*, and *Methylobacterium-Methylorubum*, respectively (Fig. S3B). Other clinically important species detected here, such as *Corynebacterium*, have also been reported to be involved in opportunistic infections (19).

*Streptococcus* was especially prevalent in Indonesia coat and trouser samples (Fig. S3A) and aligns with previous observations of *S. aureus* enrichment after laundering (20). Its reported prevalence in public and household washing machines suggests possible bacterial transfer between loads, an undesirable event for an opportunistic species (8, 20, 21). However, we note that the abundances reported here are relative, and quantification of exact viable bacterial loads will be needed to evaluate actual risks of contamination. Nevertheless, the use of clothing detergent that can reduce the proliferation of these species should be considered in a shared facility.

**Fig 5:**
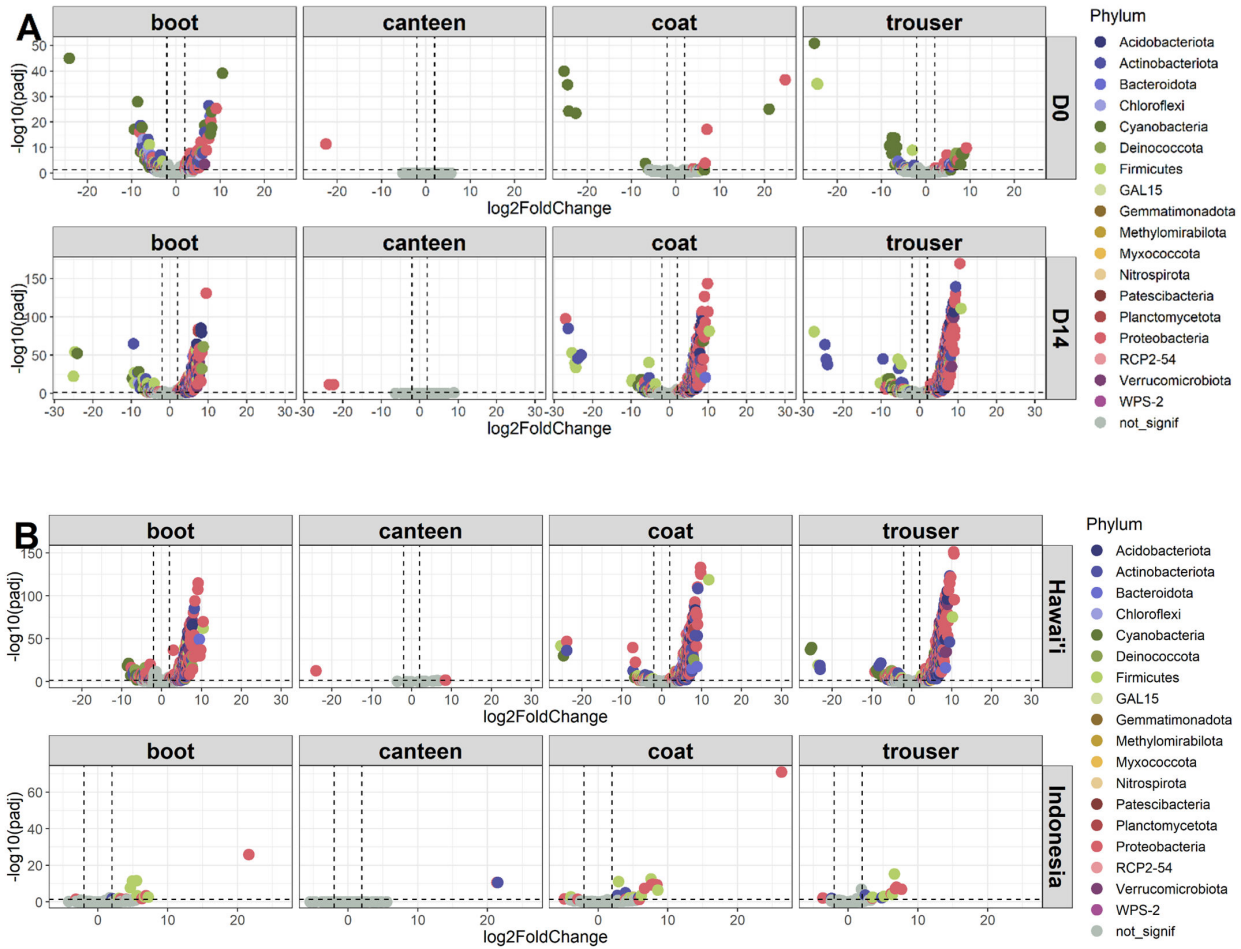
Differential abundance analyses of ASVs. DESeq2 was used to compare differentially abundant ASVs between (A) study sites and (B) timepoints for each gear type. (A) Significantly differential ASVs between study sites were compared across both D0 and D14. A positive log_2_ fold change represents ASVs that were enriched in Hawai’i samples, and a negative log fold change represents ASVs that were enriched in Indonesia samples. (B) Significantly differential ASVs between timepoints were compared for Indonesia and Hawai’i samples. A positive log fold change represents ASVs that were enriched in D14, and a negative log fold change represents ASVs that were enriched in D0. Each point represents an ASV, and colors correspond to different phyla. Horizontal dotted lines correspond to a cutoff *P*-value of 0.05, and vertical dotted lines correspond to absolute log_2_ fold change values of 2.

Differential abundance analysis was conducted to determine the number of significantly different amplicon sequence variants (ASVs) between study sites and timepoints (Fig. 5; Table 2). Few ASVs were found to be significantly differentially abundant in canteen samples regardless of study site or sample timepoint (Table 2; Tables S1 to S4). Notably, a high number of significantly differential ASVs were found at D14 (boot: 439, canteen: 3, coat: 482, and trouser: 500) compared to D0 (boot: 87, canteen: 1, coat: 57, and trouser: 100) for Hawai’i samples (Table S2). A majority of these ASVs comprised *Proteobacteria* (boot: 206, coat: 207, and trouser: 216), *Acidobacteriota* (boot: 71, canteen: 0, coat: 75, and trouser: 75), and *Actinobacteriota* (boot: 67, canteen: 0, coat: 76, and trouser: 82). At D14, the number of significantly differential ASVs was higher in Hawai’i samples (boot: 471, canteen: 0, coat: 479, and trouser: 505) compared to Indonesia samples (boot: 96, canteen: 3, coat: 90, and trouser: 98) (Table S4). In contrast, few ASVs were found to have significantly increased across time in Indonesia samples with an average of 1.5 and 11.25 ASVs across samples at D0 and D14, respectively (Table S3).

**TABLE 2:** Number of significantly enriched ASVs for each study site or timepoint comparison across all four gear types^*a*^

Comparison between study sites			
	Gear type	Indonesia	Hawai’i
D0	Boot	128	106
	Canteen	2	0
	Coat	6	19
	Trouser	38	32
D14	Boot	96	471
	Canteen	3	0
	Coat	90	479
	Trouser	98	505
**Comparison between timepoints**			
	**Gear type**	**D0**	**D14**
Indonesia	Boot	2	14
	Canteen	0	1
	Coat	2	19
	Trouser	2	11
Hawai’i	Boot	87	439
	Canteen	1	3
	Coat	57	482
	Trouser	100	500

^*a*^ Results from differential abundance analyses capturing the number of significantly enriched ASVs between study sites or timepoints.

As *Acinetobacter baumannii* has been widely reported to be a cause of military wound infections (22, 23), we assessed the presence of this species in a subset of samples (*n* = 45) using quantitative PCR (qPCR). We selected samples across all locations and gear types and included samples that were negative and positive for *Acinetobacter* based on the 16S rRNA sequencing data. We detected the presence of *A. baumannii* (via qPCR) in 30 samples that were *Acinetobacter*-positive (via 16S rRNA sequence), while *A. baumannii* was not detected in five out of six samples that were *Acinetobacter*-negative (Fig. S4). This is likely attributable to the higher sensitivity of the species-level qPCR assay. Furthermore, *A. baumannii* was not detected by qPCR in nine *Acinetobacter*-positive samples, indicating the presence of other *Acinetobacter* species. Overall, this assay confirmed the presence of an opportunistic species on military gear and suggests the need for future investigation at greater taxonomic and gene-level resolutions through other targeted molecular approaches.

Lastly, questionnaires were given to each participant at the end of the study to record water intake patterns and other health-relevant observations (Table S5). However, minimal occurrence of adverse symptoms was reported and, therefore, statistical associations between microbiome data and questionnaire data were not plausible due to categorical imbalances in symptom occurrence and severity scores.

### Predicting potential risk factors across the gear microbiome

**Fig 6:**
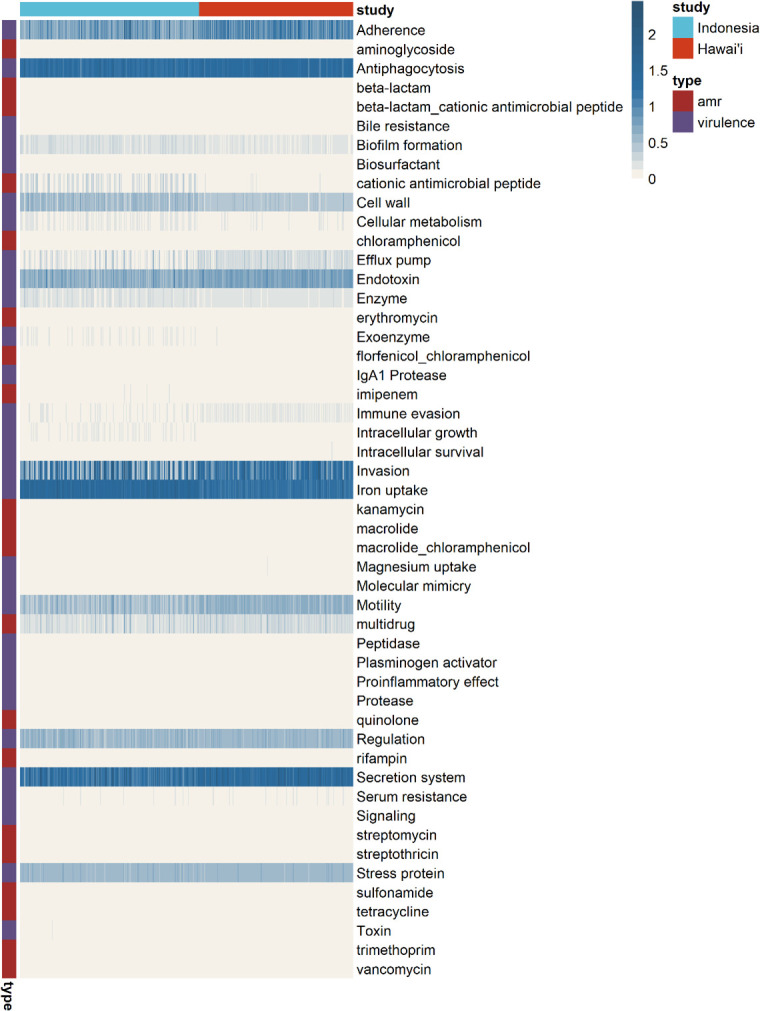
Relative abundance distribution of predicted antibiotic resistance and virulence genes across samples. Each column corresponds to a single sample and each row corresponds to either an antibiotic class or a virulence category. Samples are annotated according to study site and functional category.

The taxonomic analyses described above broadly demonstrate the enrichment of genera containing potentially opportunistic pathogenic species, such as *Acinetobacter* and *Pseudomonas*. The transfer and persistence of these species could be detrimental as they harbor properties such as biofilm formation and antibiotic resistance that influence wound progression, care, and treatment decisions. As it is not possible to obtain functional information from amplicon sequencing, PICRUSt2 was used to predict potential antimicrobial resistance and virulence burden that may be present within the military gear microbiome. The functional predictions include antimicrobial resistance genes involved in multidrug resistance and cationic antimicrobial peptide resistance and virulence genes involved in adherence, antiphagocytosis, invasion, iron uptake, and secretion systems (Fig. 6). Some of the species that were predicted to harbor antimicrobial resistance genes were *Staphylococcus*, several *Methylophilaceae* species, *Pseudomonas*, *Alcaligenes*, and *Acinetobacter* (Fig. S5A). Moreover, *Rahnella*, *Pseudomonas*, *Pseudacidovorax*, *Massilia*, and *Staphylococcus* were predicted to contribute highly toward virulence (Fig. S5B).

Although functional inference from amplicon data is limited by biases in databases and the ability to delineate strain-specific properties (24), these preliminary findings align with previous studies that demonstrated the potential of materials associated with the human body to serve as vectors for infectious agents in both healthcare (25, 26) and occupational waste control environments (27), leading to efforts in improving sanitation and disinfection practices on surfaces such as protective personal equipment (28–30). However, unlike a traditional hospital environment, continuous disinfection is not practical in an operational military environment. Instead, measures such as modifying hygiene practices and innovation of antibacterial materials could be adopted.

Advancements in textile innovation in the healthcare industry have been developed to minimize the acquisition and transmission of pathogens within hospitals and into the community. This includes the impregnation of metal or metal oxide nanoparticles with antibacterial properties such as silver and titanium dioxide into various fabrics (31). Evidence presented from a clinical trial demonstrated that patients with burns and lesions had reduced levels of nosocomial pathogens (*S. aureus* and *A. baumannii*) when using zinc oxide-coated fabrics compared to standard fabrics (32). Thus, our results provide an opportunity to leverage these innovations in the military space to minimize the risks of contamination and infection. The data presented here, describing the background bioburden surrounding the warfighter in operational environments, provide critical information on the potentially infectious targets that can be prioritized for future antimicrobial materials design.

### Conclusion

In conclusion, our study presents novel information on the military gear microbiome in real-world settings, highlighting spatiotemporal differences along with potential risk factors that exist around the warfighter. Despite the limited taxonomic resolution obtained from amplicon sequencing, our results preliminarily suggest the need for modified hygiene practices during military training along with improvements in antimicrobial designs for materials used to manufacture military gear. Future studies that integrate dense longitudinal sampling, collection of health-relevant metadata, and the surveillance of microbial signatures at greater resolution, along with other microbial species such as fungi and viruses, are necessary to fully elucidate risk factors that could be present. Such efforts would further inform future interventions that support the health and safety of injured U.S. service members.

## MATERIALS AND METHODS

### Study design and sample collection

Specimens were collected from the gear and environment of students participating in the JOTC conducted in Oahu, Hawai’i, and from active-duty service members stationed on Oahu deployed to Indonesia for a field exercise. Those participating in the joint training field courses and exercises were briefed on the study, and participants who consented to gear swabs were assigned an identification number. Materials associated with service members, including uniforms and boots, were swabbed using Puritan DNA-free swabs and Teknova PCR-certified water (5 mL vial). Swabs were dipped in DNA-free water (5 mL vial) and depressed against the side of the tube to remove excess liquid. A maximum area of 4″ × 4″ was sampled. For larger surface areas, an overlapping S-shaped pattern was utilized for the collection. Water samples were obtained from canteens and/or camelback at both locations. Uniforms were sampled at the sleeve cuff of the dominant hand, front abdominal area, lower back area, and trouser waistline/pocket area of the dominant hand. Boot samples were collected from the upper surface and sole. A de-identified questionnaire was administered to collect the time of canteen filling, the time of the most recent canteen use, and the presence of any enteric symptoms (diarrhea, stomach aches, vomiting, and nausea) experienced by the participants. Baseline samples were taken at the start of training (± 5 days) and 2 weeks later. Swabs and water samples were stored at −80°C prior to analysis.

### Specimen extraction, library preparation, and sequencing

Specimens from participants who completed all sampling locations from both timepoints were selected for microbiome profile processing. This included 28 participants from Hawai’i and 33 participants from Indonesia resulting in a total of 488 sequenced samples. Swab tips were transferred to microcentrifuge tubes containing ZR Bashing Beads (Zymo Research). Seven hundred fifty microliters of ZymoBIOMICS Lysis solution was added to the tube followed by bead beating on the FastPrep-24 system (MP Bio), using three cycles of 40 s at 6 m/s and 5-minute rest. DNA was purified from the samples using the ZymoBIOMICS DNA Miniprep Kit according to the manufacturer’s recommendations (Zymo Research) and eluted in 75 µL of ZymoBIOMICS DNase/RNase-Free Water.

A total of 600 µL of each canteen water sample was added to a Lysing Matrix F tube (MP Bio) containing 150 µL of Buffer ATL and Reagent DX (Qiagen), followed by bead beating on the FastPrep-24 system using one cycle of 40 s at 6 m/s. Following bead beating, tubes were centrifuged, and 400 µL of supernatant was used as input into the QIamp UCP Pathogen Mini kit (Qiagen). Standard manufacturer’s recommendations were followed, and samples were eluted in buffer AVE.

The 16S rRNA region was amplified using primers targeting the V3-V4 region

(forward primer: 5′-TCGTCGGCAGCGTCAGATGTGTATAAGAGACAGCCTACGGGNGGCWGCAG-3′

reverse primer: 5′-GTCTCGTGGGCTCGGAGATGTGTATAAGAGACAGGACTACHVGGGTATCTAATCC-3′). Sequence library preparation was performed using a two-step PCR approach, following the “16S Metagenomic Sequencing Library Preparation” guide (Illumina, part # 15044223 rev B). Completed library pools were assessed on the Tapestation 4200 (Agilent). Sequencing was performed on the Illumina MiSeq using the 600-cycle v3 reagent kit.

### 16S rRNA analysis

Ninety-five samples were excluded due to low read counts or poor sequence quality. Downstream analyses were conducted on 208 Indonesia samples (62 boot, 32 canteen, 51 coat, and 63 trouser) and 179 Hawai’i samples (55 boot, 15 canteen, 53 coat, and 56 trouser). Rarefaction analysis was subsequently conducted to determine read cutoffs. Read cutoffs were extrapolated from an asymptotic curve, whereby the selected cutoff captured stabilizing diversity while retaining as many samples as possible. Canteen samples had the least number of reads and a 550-read cutoff was applied. A 2,000 read cutoff was applied to boot, coat, and trouser samples. Samples below the read threshold were not analyzed downstream. A total of 4 boot samples, 11 coat samples, and 1 trouser sample were discarded due to reads below the defined threshold. 16S rRNA sequencing data were analyzed using QIIME2. Amplicon sequence variants were obtained using DADA2. Taxonomic assignments were based on the SILVA database (33).

Functional predictions of microbiome samples and taxonomic contributions were carried out using PICRUSt2 (24). PICRUSt2 aligns and places sequences (ASVs) into a reference tree containing 20,000 full-length 16S rRNA genes and infers gene family copy numbers based on a database of reference genomes and gene families. The abundances of gene families per sample are estimated based on the predicted gene content per ASV and ASV abundances. PICRUSt2 supports annotation against gene family databases such as Kyoto Encyclopedia of Genes and Genomes (KEGG) orthologs, Cluster of Orthologous Groups, and Enzyme Commission numbers. In our analysis, gene families were assigned to KEGG Orthologs (KOs) and data tables of per-sample predicted metagenomic contributions and corresponding ASV contributions were generated. Annotations of antimicrobial resistance genes were mapped directly from the KEGG database (34), while virulence gene annotations were obtained from a previously curated database consisting of virulence-relevant KOs [Pattaroni et al., 2018 (35); Custom Virulence Factor Database for 16S metagenomics]

### Statistical analysis

Downstream biostatistical analysis and visualization were conducted in R (version 4.2). Alpha (observed ASVs and Shannon index) and beta diversity (MDS of Bray-Curtis distances) measurements and taxonomic relative abundances were obtained using phyloseq (36). PERMANOVA analysis was carried out using the vegan package (37). DESeq2 was used for differential abundance analysis (38), and heat trees were visualized using metacodeR (39). Wilcoxon rank-sum tests were used for bivariate comparisons followed by FDR adjustment when applied to multiple comparisons.

### Quantitative-PCR analysis

Quantification of *A. baumannii* was carried out using quantitative PCR on a subset of 45 samples using the Applied Biosystems 7500 Fast Real-Time PCR System (Thermo Fisher Scientific). Each reaction mixture contained 12.5 µL of qPCR Master Mix (iTaq Universal SYBR Green Supermix; Bio Rad), 1 µL each of 10 µM forward (5′-CATTATCACGGTAATTAGTG-3′) and 10 µM reverse primer (5′-AGAGCACTGTGCACTTAAG-3′), 8.5 µL of water, and 2 µL of template. A standard curve was generated using 10-fold serial dilutions of *A. baumannii* strain 2208 (ATCC 19606D-5) genomic DNA. *A. baumannii* species-specific primers targeted the internal transcribed spacer region between the 16S and 23S rRNA gene (40). Cycling conditions were as follows: initial denaturation at 95°C for 5 minutes; 35 cycles at 95°C for 20 s, 55°C for 45 s, and 72°C for 30 s, followed by a melting curve, gradually increasing from 65°C to 95°C. Samples were analyzed in triplicate. Samples, whereby CT values were undetermined, were labeled as *A. baumanii*-negative.

## Data Availability

The raw data sets generated in this study are not publicly available due to sensitivities regarding military service member cohorts but are available from the authors on reasonable request and in accordance with applicable regulations and data usage agreements.

## SUPPLEMENTAL MATERIAL

Tables S1 to S5 aem.01176-23-s0001.xlsx Supplemental tables.

Fig. S1 to S5 aem.01176-23-s0002.docx Supplemental figures.

ASM does not own the copyrights to Supplemental Material that may be linked to, or accessed through, an article. The authors have granted ASM a non-exclusive, world-wide license to publish the Supplemental Material files. Please contact the corresponding author directly for reuse.
