# Supplementary material for: The military gear microbiome: risk factors surrounding the warfighter
Source: Appl Environ Microbiol. 2024 Jan 3;90(1):e01176-23. doi: 10.1128/aem.01176-23 (PMC10807412; doi:10.1128/aem.01176-23)
Supplement: Fig. S1 to S5 — Supplemental figures. [file aem.01176-23-s0002.docx]

**
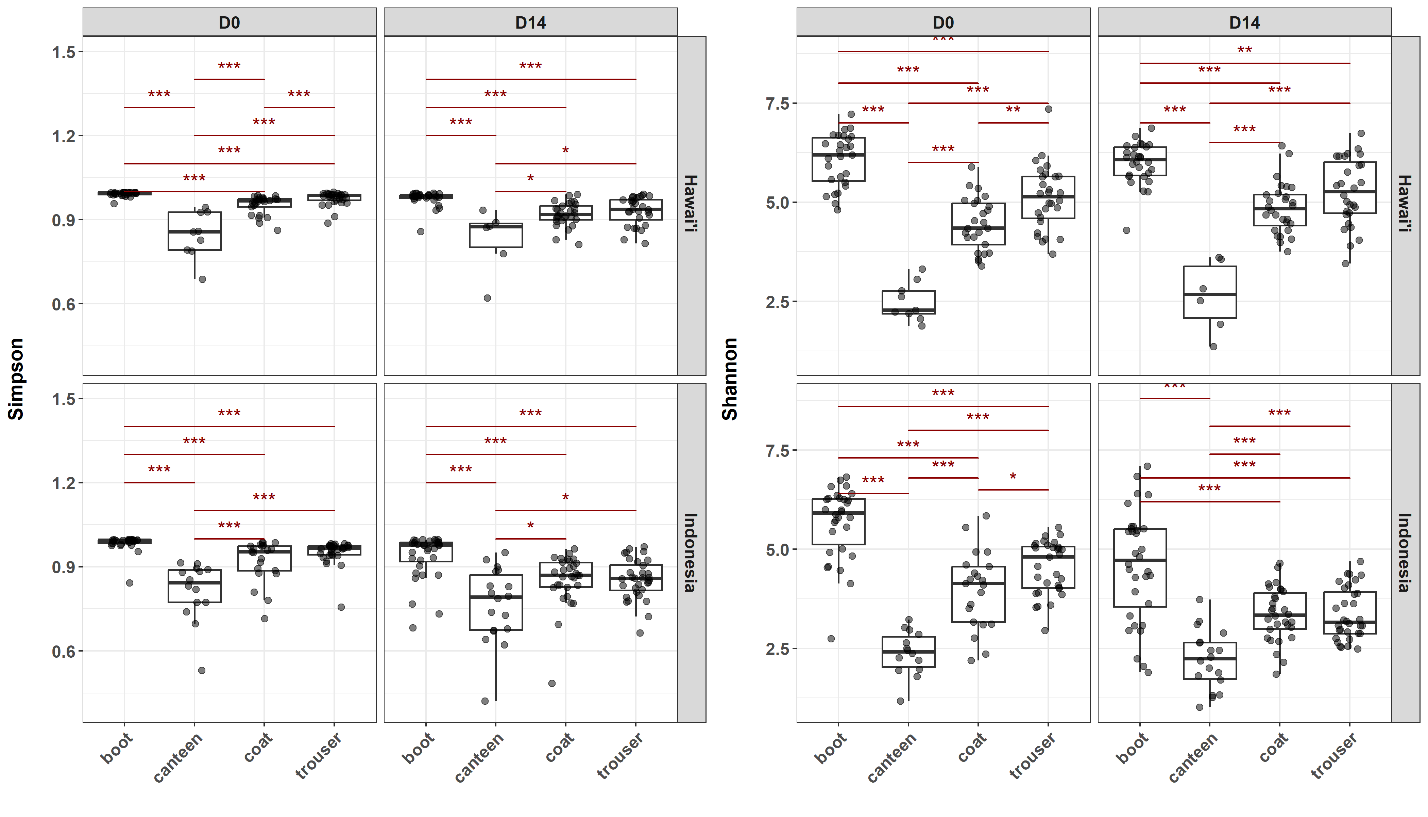
**

**A**

**B**


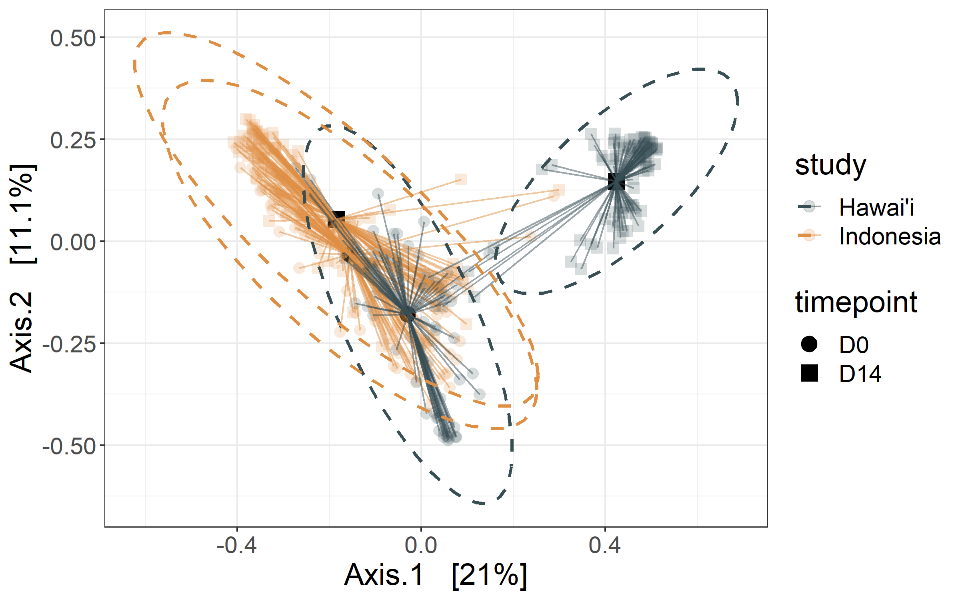


Figure S1. Microbial diversity of gear samples: A) Alpha diversity measurements between gear type across study sites and timepoints. B) MDS plot visualizing compositional differences between all samples collected in this study. Samples are colored according to study site, and symbols represent timepoints.


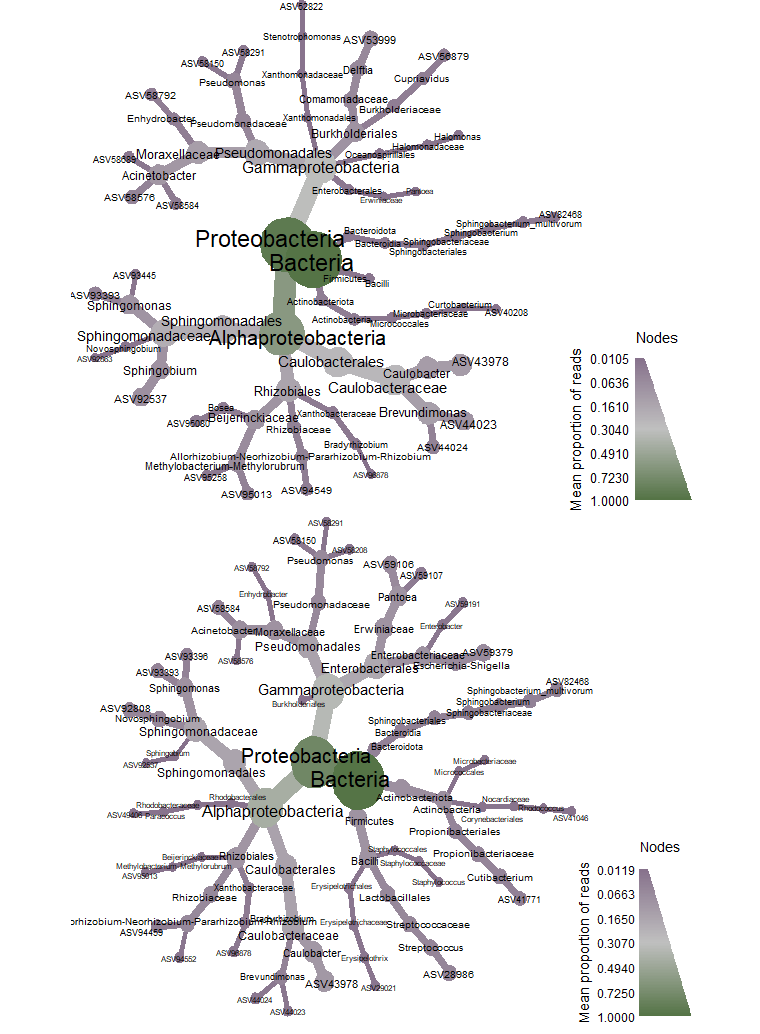


**A**

**B**

Figure S2. Heat trees visualizing mean proportion of reads: Reads assigned to each taxonomic lineage rank as identified in A) Indonesia and B) Hawai’i canteen samples across both timepoints. Both node size and color intensity represent the mean proportion of reads.


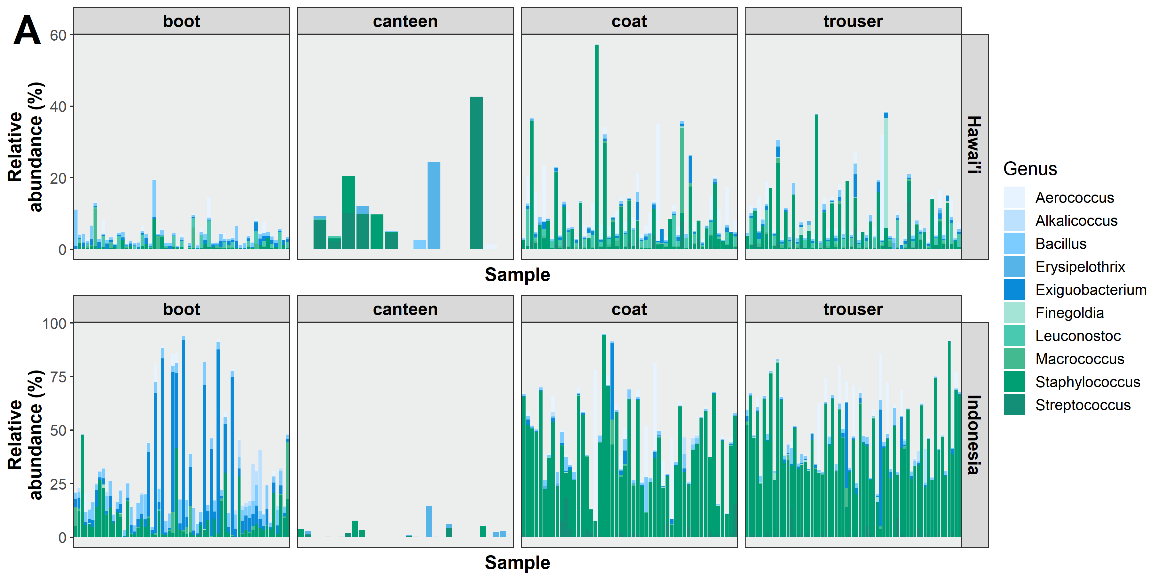

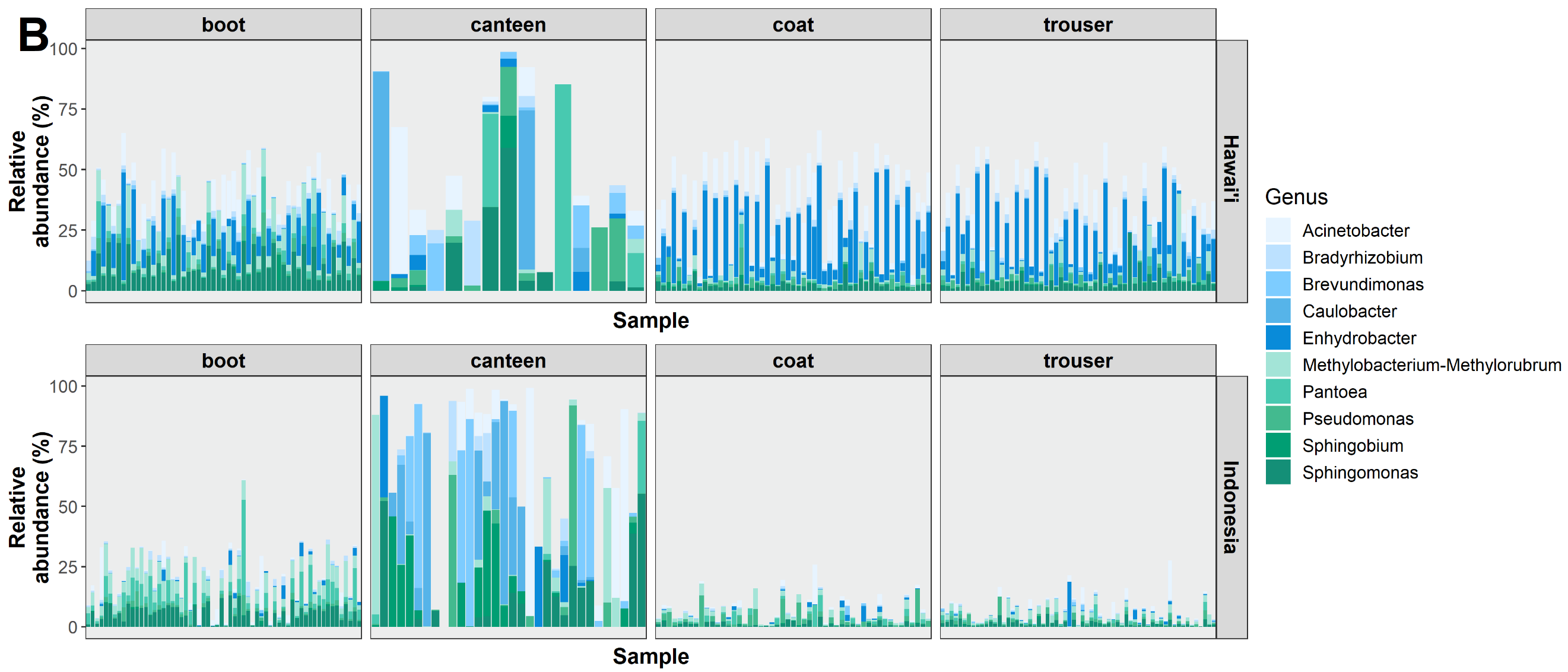


Figure S3. Abundant species present across gear samples: The top 10 most abundant A) *Firmicutes* and B) *Proteobacteria* genera present across gear samples and sampling location. Each column corresponds to a single sample and colors represent a genus.


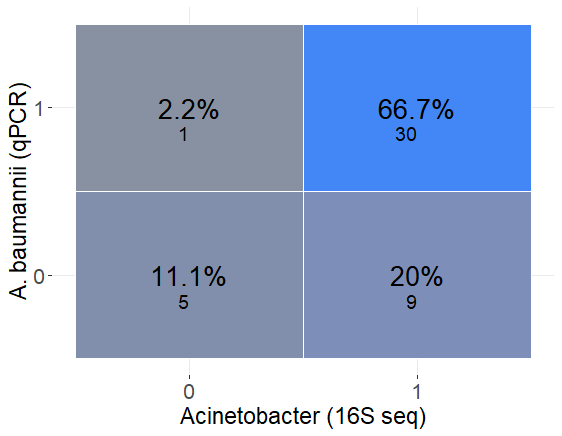


**A**

**B**


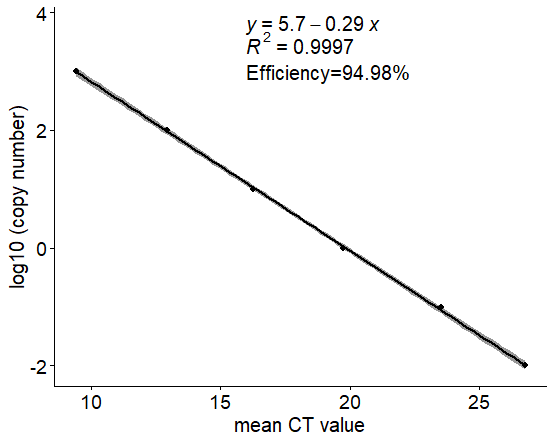


Figure S4. Quantitative PCR analysis of *Acinetobacter baumannii* across 45 gear samples. A) qPCR standard curve generated using genomic DNA from *A. baumannii* strain 2208 (ATCC 19606D-5). B) Classification matrix comparing detection of *Acinetobacter* through 16S rRNA sequencing with *Acinetobacter baumannii* detection using qPCR.

**A**

**B**


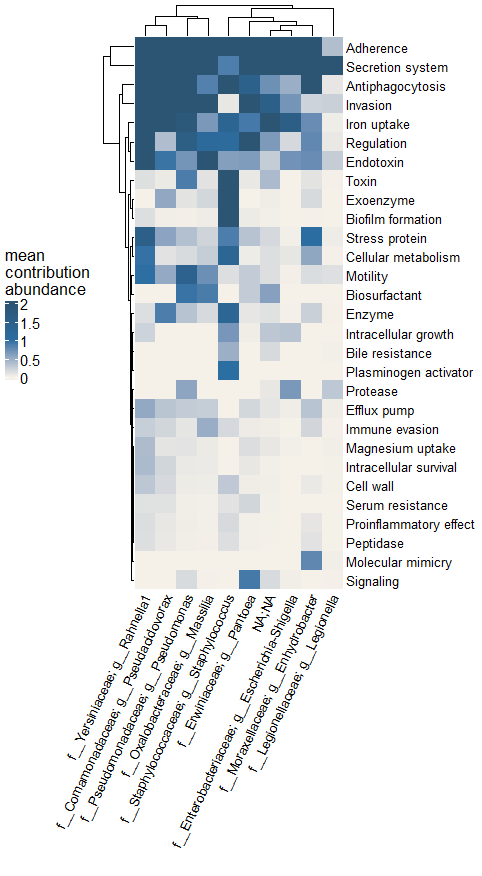

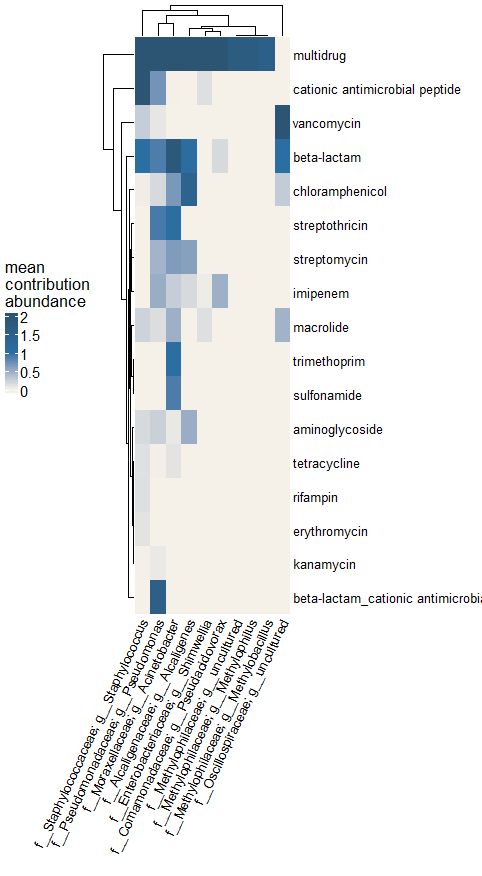


Figure S5. Predicted microbial contributors to antimicrobial resistance and virulence genes: Top 10 contributing taxa to A) antimicrobial resistance genes and B) virulence factors as predicted using PICRUSt2. Each column represents a taxonomic species, and each row represents either an antibiotic class or a virulence category.
